# Comparison on the Effects and Safety of Tualang Honey and Tribestan in Sperm Parameters, Erectile Function, and Hormonal Profiles among Oligospermic Males

**DOI:** 10.1155/2014/126138

**Published:** 2014-11-19

**Authors:** Shaiful Bahari Ismail, Mohd. Bustamanizan Bakar, Nik Hazlina Nik Hussain, Mohd Noor Norhayati, Siti Amrah Sulaiman, Hasnan Jaafar, Samsul Draman, Roszaman Ramli, Wan Zahanim Wan Yusoff

**Affiliations:** ^1^School of Medical Sciences, Universiti Sains Malaysia, Health Campus, 16150 Kubang Kerian, Kelantan, Malaysia; ^2^International Islamic University, Bandar Indera Mahkota, 25200 Kuantan, Pahang, Malaysia; ^3^Hospital Raja Perempuan Zainab II, 15200 Kota Bharu, Kelantan, Malaysia

## Abstract

*Introduction.* This study aims to evaluate the effectiveness of Tualang honey on sperm parameters, erectile function, and hormonal and safety profiles. *Methodology.* A randomized control trial was done using Tualang honey (20 grams) and Tribestan (750 mg) over a period of 12 weeks. Sperm parameters including sperm concentration, motility, and morphology were analyzed and erectile function was assessed using IIEF-5 questionnaire. Hormonal profiles of testosterone, FSH, and LH were studied. The volunteers were randomized into two groups and the outcomes were analyzed using SPSS version 18. *Results.* A total of 66 participants were involved. A significant increment of mean sperm concentration (*P* < 0.001), motility (*P* = 0.015) and morphology (*P* = 0.008) was seen in Tualang honey group. In Tribestan group, a significant increment of mean sperm concentration (*P* = 0.007), and morphology (*P* = 0.009) was seen. No significant differences of sperm concentration, motility, and morphology were seen between Tualang honey and Tribestan group and similar results were also seen in erectile function and hormonal profile. All safety profiles were normal and no adverse event was reported. *Conclusion.* Tualang honey effect among oligospermic males was comparable with Tribestan in improving sperm concentration, motility, and morphology. The usage of Tualang honey was also safe with no reported adverse event.

## 1. Introduction

Infertility is a disease of the reproductive system defined by the failure to achieve a clinical pregnancy after 12 months or more of regular unprotected sexual intercourse [1]. Infertility is a global health problem affecting couples worldwide with estimation of about 8 to 12% of them experiencing some form of infertility during their reproductive life [2]. Causes of infertility include male, female, couple factors, and unexplained causes. The male factor is usually related to sperm abnormality, while female factors are related ovarian dysfunction and tubal pathology [3].

Male infertility involves a complex aetiology. There are many factors contributing to male infertility such as structural abnormality, hormonal imbalance, previous infection, environmental factor, immunological factor, genetic factor, systemic disease, erectile function, spermatogenic dysfunction, and idiopathic. An evaluation related to spermatogenesis was done by many researchers using a variety of treatment models to treat infertility in male [4].

Measurements of sperm quality and quantity such as sperm concentration, sperm motility, and sperm morphology of seminal fluid were included in the conventional semen analysis. Normal values of semen parameters issued by the World Health Organisation (WHO) are generally used as the reference values [5].

Various researches were carried out for treatment of oligospermic males using different types of medicines, hormones, vitamins, herbs, or minerals. In relation to that, many experimental procedures were employed on animals with various types of materials to determine their effectiveness on spermatogenesis postulated for human use [4].

### 1.1. Tribestan (*Tribulus terrestris*)

Usage of* Tribulus terrestris* (herbs) for enhancement of spermatogenesis has been reported many years ago. Studies of* Tribulus terrestris* in animals have showed intensification of spermatogenesis [6] and increase of testosterone level [7]. Besides,* Tribulus terrestris* also has antioxidant activity [8], antimicrobial activity [9], and antihypertensive and vasodilator effect [10]. The first standardized preparation of* Tribulus terrestris* was by Sopharma, Bulgaria; “Tribestan” was well established three decades ago and widely used in clinical trials. Tribestan has been recommended for the treatment of impotent and libido disorders in male [11].

### 1.2. Honey

Medical use of honey was documented in many ancient written records among Egyptians, Assyrians, Chinese, Greeks, and Romans. The Holy Quran and Bible also describe the importance of honey in human's life and there is one surah in Quran which is named after the bees. Honey, naturally produced from the belly of bees, contains not less than 181 different compounds including simple and complex sugars, organic acids, minerals and trace elements, vitamins, amino acids, proteins (mainly enzymes), lipids (simple, complex, and wax), plant flavours and colouring materials, hydrocarbons, hormones, pollens, and microorganisms (yeast) [12].

Tualang honey is one of several types of honey found in Malaysia besides Gelam and Akasia honey. Tualang honey was named after the Tualang tree (*Koompassia excelsa*) where* Apis dorsata* bees build their nest to produce honey. The Tualang tree can be found in the lowland rainforest of southern Thailand, north-eastern Sumatra, and also Malaysia [13]. About 14–18 compounds in Tualang honey have antioxidant properties [14].

Local research teams have evaluated the effect of Tualang honey in various studies. In animal studies, Tualang honey has been used in wound treatment [15–17] and as apoptosis inducer [18] while, in human, it has been used for postmenopausal treatment [18–22], scar treatment [23], and acute respiratory symptoms [24]. Mohamed et al. have used Tualang honey in spermatogenesis studies in healthy rats and rats exposed to cigarette smoke [25]. The studies reported that Tualang honey enhanced spermatogenesis in both rat samples leading to increase in the sperms and the spermatic counts. The testosterone hormone level also showed significant improvement although no changes were seen in follicular stimulating hormone and luteinizing hormone level [25]. The quality of sperms too improved with Tualang honey and the percentage of abnormal sperms reduced significantly [25, 26]. These results were probably due to the antioxidant protective effect of Tualang honey as supported by Mohamed et al. who reported that Tualang honey has large amount of antioxidant properties derived from its phenolic constituents [14].

### 1.3. Justification of the Study

There were numerous studies conducted all over the world aiming to evaluate the medicinal value of honey. However, there was no reported study on the effect of honey in improvement of sperm concentration, motility, and morphology in human as well as on erectile function and male hormone. Therefore, the main study objective is to determine the effectiveness of Tualang honey in improving sperm parameters, erectile function, and hormonal profiles among oligospermic males. Data from this study is essential for future application on the use of honey for the treatment of male infertility and erectile function.

## 2. Materials and Methods

This study is an open-label randomized control trial among male patients attending infertility clinic at two centres that were Universiti Sains Malaysia Hospital, Kubang Kerian, Kelantan, and International Islamic University of Malaysia, Kuantan, Pahang, from June 2010 until December 2012. Inclusion criteria were being married, having alive wife/wives, being of age > 20 and <55 years old, and having sperm count between 5 and 20 × 10^6^/mL, and being literate. Exclusion criteria were having anatomical penile deformities, history of undescended testes, history of vasectomy or orchidectomy, major uncontrolled psychiatric disorders, history of alcohol or drug abuse, history of major hematological, renal, hepatic, or bleeding disorder, stroke or myocardial Infarction within 6 months, systolic blood pressure < 90 or >170 mmHg and/or diastolic blood pressure < 50 or >100 mmHg, abnormal full blood count, renal profile, or liver function test, and uncontrolled diabetes mellitus (HbA1c > 7%) and using herbs or drugs that could contain androgenic activity in the last three months.

Study sample size was calculated using PS power and sample size calculation software version 3 for all objectives with a power of 90% and statistical significance (*α*) of 0.05. Considering the nonresponse rate of 10%, 34 patients were recruited for each arm. They were assigned to either Tualang honey or Tribestan group via computer block randomization based on their identification card number.

### 2.1. Study Tools

*(i) Sperm Analysis.* Sperm analysis was done according to the fourth WHO manual sample collection and delivery. This investigation was sent and analysed either in Universiti Sains Malaysia Hospital or in International Islamic University of Malaysia. The respondent underwent three days abstinence before semen collection. The seminal fluid was collected in a special sterile plastic container and it was sent to the respective lab within one hour after masturbation. Sperm analysis was done during second visit and it was repeated during the fourth visit after completion of 12-week study. The sperm parameters used in this study are as showed in Table 1.

*(ii) Blood Investigations.* The blood investigations done in this study were full blood count (FBC), renal function test (RFT), liver function test (LFT), total serum testosterone, follicular stimulating hormone, and luteinizing hormone. About 10 mL of the respondent's blood was taken from antecubital fossa and sent either to Gribbles Laboratory in Kuantan, Pahang, or to Kota Bharu, Kelantan, for analysis. Blood samples were taken during the second and fourth visits.

*(iii) Questionnaire.* The questionnaire used in this study was International Index of Erectile Function (IIEF-5). The IIEF-5 questionnaires contain five questions for patient self-assessment to detect and classify the severity of erectile dysfunction. Erectile dysfunction severity was classified into the following five categories based on IIEF-5 scores: no erectile dysfunction (22–25), mild erectile dysfunction (17–21), mild to moderate erectile dysfunction (12–16), moderate erectile dysfunction (8–11), and severe erectile dysfunction (5–7). IIEF-5 was given to the respondents in the second and fourth visits. A validated IIEF-5 Malay version [27] was used in this study as all the respondents understand Malay language well.

*(iv) Physical Examination.* During physical examination, the respondents were examined for height, weight, blood pressure, pulse rate, testicular volume, cardiorespiratory, and abdominal examination. Height and weight were measured using Seca scale with the height being recorded in meter (2 decimals) and the weight being recorded in kilogram (1 decimal). Blood pressure was measured using calibrated manual sphygmomanometer and pulse rate was counted manually within 60 seconds. Testicular size was measured using orchidometer. These procedures were done during the second and fourth visits.

### 2.2. Study Intervention

*(i) Tualang Honey.* Tualang honey used in this study was collected from FAMA Kedah and sterilized by gamma radiation done before packing in a sachet form. The respondents in Tualang honey group were given 20 grams of Tualang honey. With an estimated adult male body weight of 75 kg, hence, 90 grams of Tualang honey was calculated. However, based on traditional local human consumption of honey of 0.2 g/kg body weight [25], an estimate of around 15 grams of honey was used. Therefore, the amount of 20 grams of honey was a reasonable dose to be taken by the respondents in this study.

*(ii) Tribestan.* Tribestan tablet was used in this study as a comparator.

### 2.3. Study Procedure

This study was conducted in accordance with Good Clinical Practice guideline and Declaration of Helsinki. Approval from Human Research and Ethics Committee for Clinical Studies of Universiti Sains Malaysia (USM) (Reference Number: USMKK/PPP/JEPeM [223.3.911]) was made.

All men who were involved in the study voluntarily participated and written informed consent was taken after a comprehensive explanation was given regarding the proposed treatment involved, nature of the therapy, anticipated benefit, and any known side effects of the therapy during screening visit (first visit). A study information sheet was given and they were allowed to withdraw from the study without penalty at any time.

During the second visit, demographic data, general conditions, and genitalia examination were done to exclude structural or other treatable causes. The respondents underwent confirmatory sperm analysis and the baseline blood investigations (FBC, RFT, and LFT) with hormonal profiles (total testosterone, FSH, and LH) were taken in early morning. The IIEF-5 questionnaire was given to the respondents in this visit. The respondents who fulfilled the criteria were randomised to either Tualang honey or Tribestan group.

The respondents in the Tualang honey group were instructed to take 20 grams of Tualang honey early in the morning 30 minutes before breakfast daily for 12 weeks. Meanwhile, the respondents in the Tribestan group were instructed to take 250 mg Tribestan for three times daily after meals for the same period of time. These medications were dispensed to the respective group during this visit with amount of medication given calculated based on the next follow-up visit.

In the third visit, compliance towards medication and medication side effects were evaluated. The respondents were required to bring back the remaining Tualang honey or Tribestan for compliance assessment. Medications were supplied to the respondents to be completed for a total of 12-week period.

In the fourth visit, all the respondents were reexamined for their general conditions and blood investigations including FBC, RFT, LFT, total testosterone, FSH, and LH were repeated. Sperm analysis was repeated and erectile function was reevaluated using IIEF-5 questionnaire.

**Figure 1:**
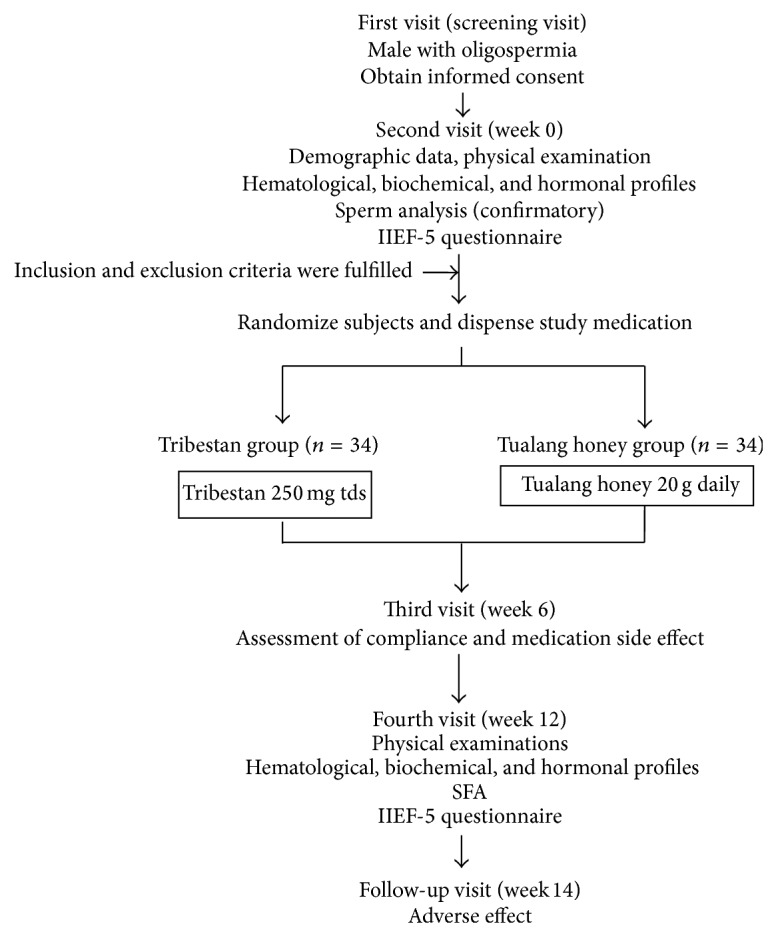
Flow chart of the study.

During the fifth visit, that is, about two weeks after completion of the study, the respondents were contacted asking on any other adverse effects.  Figure 1 showed flow chart of the study.

### 2.4. Statistical Analysis

The data were analysed using Statistical Package for Social Sciences (SPSS) 18.0 under license of USM, Malaysia. In descriptive analysis, mean (SD, standard deviation) was used to describe continuous variables while frequency and percentage (%) were used to describe categorical. Sociodemographics and baseline medical characteristics were compared between two groups using chi-square tests for categorical data and ANOVA for numerical data. Paired *t*-test was used for comparisons between pre- and postinterventions in sperm concentration, motility, and morphology of Tualang honey and Tribestan groups. ANOVA was used for comparison of postintervention outcomes of sperm concentration, motility and morphology, erectile function, and hormonal profiles between two groups. After potential confounders were controlled, difference in sperm concentration, motility and morphology, erectile function, and hormonal profiles postintervention between two groups were analysed using ANCOVA analysis. Repeated measure ANOVA was used to analyse safety profiles between two groups pre- and postintervention. *P* value < 0.05 was used to denote statistical significance.

## 3. Results

### 3.1. Sociodemographic and Baseline Medical Characteristics

Mean age of the respondents in Tualang honey and Tribestan group was 34.0 and 34.9 years, respectively. Most of the respondents in this study were Malays with only six respondents in Tualang honey group and three respondents in Tribestan group who were non-Malays. More than 70% of the respondents in both groups were nonsmokers. The respondents in Tualang honey group and Tribestan group had mean systolic blood pressure and diastolic blood pressure in normotensive range. Nevertheless, mean body mass index (BMI) of the respondents in both groups was overweight (BMI > 25 kg/m^2^) with 27.4 kg/m^2^ in Tualang honey group and 26.2 kg/m^2^ in Tribestan group.

Mean sperm concentration preintervention in Tualang honey group was 12.4 × 10^6^/mL and 12.9 × 10^6^/mL in Tribestan group. Meanwhile, sperm motility and sperm morphology in Tualang honey group were 38.3% and 65.1%, respectively, as compared to Tribestan group which were 40.4% and 63.0%. However, there were no statistical significant difference in sperm parameters (sperm concentration, sperm motility, and sperm morphology) between both groups (Table 2).

Mean IIEF-5 score among respondent in both groups showed mild erectile dysfunction (score 17–21) with 20.1 score in Tualang honey group and 20.8 score in Tribestan group. However, the difference was not significant. Grading of severity of erectile dysfunction among the respondents between Tualang honey and Tribestan group showed statistically significant difference (*P* = 0.041).

Mean hormonal profiles of LH, testosterone, and FSH among the respondents in both groups were quite similar. The values of LH, testosterone, and FSH were 6.0 IU/L, 15.1 nmol/L, and 6.4 IU/L, respectively, in Tualang honey group and 5.1 IU/L, 15.5 nmol/L, and 7.3 IU/L, respectively, in Tribestan group.

### 3.2. Sperm Parameters

ANOVA showed no significant difference in sperm concentration, motility, and morphology between Tualang honey and Tribestan groups postintervention; and ANCOVA showed no significant difference in sperm concentration, motility, and morphology between Tualang honey and Tribestan groups when age, smoking status, and baseline sperm concentration and motility or morphology were included in the model (Table 3).

There were statistically significant differences between pre- and postintervention of sperm concentration (*P* < 0.001), motility (*P* = 0.015), and morphology (*P* = 0.008) in Tualang honey group. However, paired *t*-test only showed statistically significant difference between pre- and postintervention of sperm concentration (*P* = 0.007) and morphology (*P* = 0.009) in Tribestan group (Table 4).

### 3.3. Erectile Function

ANOVA showed no significant difference in IIEF-5 score between Tualang honey and Tribestan groups postintervention; and ANCOVA showed no significant difference in IIEF-5 score between Tualang honey and Tribestan when age, smoking status, SBP, DBP, total testosterone, and baseline IIEF-5 score were included in the model (Table 5).

### 3.4. Hormonal Profiles

ANOVA showed no significant difference in hormonal profile, that is, luteinizing hormone, total testosterone, and follicular stimulating hormone between Tualang honey and Tribestan groups' postintervention. Meanwhile, ANCOVA showed no significant difference in hormonal profile between Tualang honey and Tribestan groups when age and baseline hormonal levels were included in the model (Table 6).

The mean LH decreased in both groups after 12 weeks but no changes was seen in mean testosterone and FSH.

### 3.5. Safety Profiles

All safety profiles of Tualang honey and Tribestan obtained from hematology, in renal and liver function in pre- and postintervention were normal ranges. Repeated measure ANOVA showed no significant difference in haematological, renal, and liver profiles between Tualang honey and Tribestan pre- and postintervention (Table 7). There were no adverse effects reported from all the respondents throughout the study.

## 4. Discussion

Various factors were involved in male infertility leading to several studies done as it impacts the health care services and the couples. The history of honey goes back long way with numerous studies being conducted all over the world aiming to evaluate the medicinal values of honey. The animal study on Tualang honey was done to see the effect on spermatogenesis and it was continued to evaluate spermatogenesis in human in this study.

### 4.1. Sociodemographic Data and Medical Characteristics

Most of the respondents were Malays as they constitute the major population in Kelantan and Terengganu. The respondents in both groups have mean of BMI ≥ 23 kg/m^2^ and were considered as overweight. This finding showed there was possibility of relationship between oligospermia and BMI. Besides, obese and overweight men also have lower sperm quality [28, 29]. The possible explanation was due to alteration in circulation of reproductive hormone concentration [29].

### 4.2. Sperm Parameters

The respondents in Tualang honey and Tribestan group had statistically significant difference in sperm parameters from pre- to postintervention. The possible explanations of these findings were antioxidant effect of Tualang honey and Tribestan. A systematic review by Ross et al. concludes that the use of oral antioxidant could improve sperm concentration, motility, and morphology in infertile male [30]. However, the standardized amount of specific oral antioxidant used needs to be explored. Analysis of Tualang honey by Mohamed et al. showed that phenolic constituent amounts in this type of honey are relatively large [14] and that will contribute to high amount of antioxidant property in Tualang honey. Animal study also showed Tualang honey had effects on oxidative stress markers and testicular histological changes [26]. In rat exposed to oxidant (cigar smoke), Tualang honey reduced testicular damage by reducing lipid peroxidation and had protective effect against abnormal sperm parameter induced by cigar smoke. It also helps to restore antioxidant system in cigar smoke exposed rat [26]. Besides, similar changes are also seen in Tribestan in terms of significant improvement of sperm concentration and morphology.* Tribulus terrestris*, the main content of Tribestan that has high antioxidant property [8] may play a role in this effect. As a result of these, there were increments in sperm parameter postintervention of Tualang honey and Tribestan groups. After comparing between the groups, there were no significant difference in all sperm parameters postintervention between Tualang honey and Tribestan group.

### 4.3. Erectile Function

The IIEF-5 score was used to evaluate erectile function of the respondents. This tool was used because it is a simple and reliable diagnostic tool for direct assessment of males' erection function [31]. The baseline mean score of IIEF-5 among respondents in both groups showed mild erectile dysfunction. However, no significant differences in IIEF-5 mean scores at baseline were observed between Tualang honey and Tribestan groups. In addition, this study showed a significant difference in grading of erectile function severity between Tualang honey and Tribestan group preintervention. About 44.1% of the respondents in Tualang honey group have mild erectile dysfunction and the remaining are normal compared to the respondents in Tribestan group with 6.2% being mild to moderate dysfunction, 18.8% being mild dysfunction, and 75.0% being normal. This result could be explained by wide range of differences in the number of respondents in each grading group leading to significance of their differences.

The use of Tribestan in treating an erectile dysfunction was started many years ago as documented in animal and human studies due to the effect of the testosterone hormone [7, 11]. However, no previous honey study has been conducted to evaluate effect on erectile function, although increase of testosterone hormone was seen in animal study [25]. From the result of erectile function postintervention, there was no significant difference between Tualang honey and Tribestan group. However, this result could not conclude whether the effect of Tualang honey on erectile function was similar to Tribestan. Further research needs to be done among erectile dysfunction men.

### 4.4. Hormonal Profiles

Many hormones were involved in spermatogenesis. Physiologically, many hormones play a role in regulating spermatogenesis especially, FSH, LH, and testosterone. FSH gives direct effect on Sertoli cells which produce nutrients, cofactors, and proteins that are needed for normal spermatogenesis progression and support transportation of spermatozoa within seminiferous tubule lumen. Meanwhile, LH acts on Leydig cells to produce testosterone hormone for growth and division of germinal cells to form spermatozoa [32]. The baseline mean levels of testosterone hormone, FSH, and LH were within normal range and no statistically significant different was seen in preintervention mean level of these hormones between both groups.

Normally, oligospermia men have low testosterone hormone with high FSH and LH level but there were studies that reported normal level of testosterone in infertile men [33]. The possible explanation of normal testosterone in infertile men was loss of germinal epithelium in testes but Leydig cells were still intact to produce normal level of testosterone [33]. In an animal study by Mohamed et al., improvements of testosterone hormone without changes in FSH and LH in the rats exposed to cigar smoke were seen after administration of Tualang honey [25]. They also explained this effect that was possibly due to Tualang honey acting locally on testes by restoring or improving function of Leydig cells. Study on Tribestan effect on hormone level among healthy person by Milanov and Taskov showed significant increase in LH and testosterone although within the normal physiological range [34]. Unfortunately, these similar changes were not seen in this study.

### 4.5. Safety Profiles

Safety profiles are crucial and essential in clinical trial settings. Basic safety profiles such as haematological profile, renal profile, and liver function were assessed in this study. Pre- and postintervention safety profiles of Tualang honey and Tribestan were in normal range. There was no significant difference seen in blood parameters between Tualang honey and Tribestan groups pre- and postintervention. Animal study in determining possible toxicity showed no acute, subacute, or chronic toxicity with the usage of* Tribulus terrestris* [35]. Potential teratogenicity and embryogenicity also showed negative results and no potential carcinogenicity [35]. Researchers have reported good tolerance and absence of side effects with Tribestan [35].

### 4.6. Complementary and Alternative Medicine in Treating Male Infertility

Recent study by Ismail et al. [36] showed* Eurycoma longifolia* improved libido and sexual performance. Surprisingly, strong impacts on sperm parameters like seminal fluid volume and sperm motility were also seen with* E. longifolia* [36]. Use of others substances like vitamin B, folate, and vitamin C did not show any significant improvement in sperm parameters [4]. There is no evidence that empirical hormonal therapies such as human menopausal gonadotrophin (HMG), human chorionic gonadotrophin (HCG), androgen, antioestrogens (clomiphene and tamoxifen), prolactin inhibitors (bromocriptine), and steroids improve male fertility except in certain conditions such as low testosterone level, hypogonadism, and hyperprolactinaemia [4]. Use of antioxidant properties may benefit selected patients but of limited use in clinical trials [4].

## 5. Conclusion

The effect of Tualang honey was comparable with Tribestan in improving the sperm parameters. Tualang honey and Tribestan showed significant difference in sperm parameters. Hormonal profile of testosterone, FSH, and LH did not show any significant difference in both groups. The safety profile of Tualang honey and Tribestan showed no significant changes in hematological, renal, or liver functions and no adverse effect was reported. Therefore, Tualang honey and Tribestan are comparable and safe to be consumed until 12 weeks in oligospermic males.

## Figures and Tables

**Table 1:** Sperm parameter reference values according to the 4th WHO manual 1999.

Semen parameters	Reference value
Volume (mL)	≥2
pH	≥7.2
Concentration (10^6^/ mL)	≥20
Motility (% motile)	≥50
Morphology (% normal)	≥15^*^

^*^From assisted reproductive technology programmes data.

**Table 2:** Sociodemographic and baseline medical characteristics.

Variable	Tualang honey (*n* = 34)		Tribestan (*n* = 32)		*P* value
	mean (SD^a^)	*n* (%)	mean (SD^a^)	*n* (%)	
Sociodemographic					
Age (years)	34.0 (4.87)		34.9 (6.89)		0.524^b^
Marriage duration (years)	4.5 (2.50)		6.1 (4.31)		0.072^b^
Number of children	0.2 (0.39)		0.3 (0.67)		0.585^b^
Number of partner	1.0 (0.17)		1.1 (0.25)		0.526^b^
Race					
Malay		28 (82.4)		29 (90.6)	0.328^c^
Non-Malay		6 (17.6)		3 (9.4)	
Education level					
Primary and secondary school		19 (55.9)		14 (43.8)	0.325^c^
College and university		15 (44.1)		18 (56.3)	
Smoking status					
Nonsmoker		24 (70.6)		23 (71.9)	0.908^c^
Smoker		10 (29.4)		9 (28.1)	
Physical examination					
SBP (mmHg)	126.2 (13.34)		121.8 (10.20)		0.137^b^
DBP (mmHg)	80.7 (10.04)		76.6 (7.49)		0.063^b^
BMI (kg/m^2^)	27.4 (3.56)		26.2 (3.49)		0.173^b^
Size of right testes (mL)	18.0 (2.90)		16.6 (3.40)		0.081^b^
Size of left testes (mL)	17.9 (2.93)		16.4 (3.75)		0.084^b^
Sperm quantity and quality					
Sperm concentration (×10^6^/mL)	12.4 (4.58)		12.9 (4.99)		0.632^b^
Sperm motility (%)	38.3 (20.25)		40.4 (23.69)		0.692^b^
Sperm morphology (%)	65.1 (23.97)		63.0 (28.30)		0.746^b^
Erectile function					
IIEF-5 score	20.1 (2.91)		20.8 (3.47)		0.362^b^
Grading of erectile function					
No dysfunction		19 (55.9)		24 (75.0)	0.041^c^
Mild dysfunction		15 (44.1)		6 (18.8)	
Mild to moderate dysfunction		0 (0.0)		2 (6.2)	
Hormonal profile					
LH (IU/L)	6.0 (7.11)		5.1 (2.79)		0.514^b^
Testosterone (nmol/L)	15.1 (4.58)		15.5 (6.60)		0.745^b^
FSH (IU/L)	6.4 (2.86)		7.3 (3.98)		0.337^b^

^a^Standard deviation.

^b^ANOVA.

^c^Chi-squared test.

**Table 3:** Sperm parameters between Tualang honey and Tribestan.

Variable	ANOVA				ANCOVA			
	Mean (SD^a^)		*F* stat^b^	*P* value	EMM^c^ (95% CI^d^)		*F* stat^b^	*P* value
	Tualang Honey	Tribestan			Tualang Honey	Tribestan		
Sperm concentration (×10^6^/mL)	31.4 (29.12)	25.3 (25.14)	0.23	0.632	30.9 (21.27, 40.53)	23.7 (13.76, 33.71)	1.18	0.281
Sperm motility (%)	48.1 (15.12)	49.8 (16.62)	0.18	0.670	47.2 (41.44, 52.93)	48.9 (42.94, 54.80)	0.18	0.670
Sperm morphology (%)	79.2 (14.80)	77.3 (10.86)	0.35	0.558	79.3 (74.53, 84.13)	77.2 (72.23, 82.16)	0.42	0.517

^a^Standard deviation.

^b^ *F* statistic.

^c^Estimated marginal mean.

^d^Confidence interval.

**Table 4:** Sperm parameters in Tualang honey and Tribestan group.

Variable	Tualang Honey			Tribestan		
	Mean (SD^a^)		*P* value	Mean (SD^a^)		*P* value
	Preintervention	Postintervention		Preintervention	Postintervention	
Sperm concentration (×10^6^/mL)	12.4 (4.58)	31.4 (29.12)	0.000	12.9 (4.99)	25.3 (25.14)	0.007^b^
Sperm motility (%)	38.2 (20.25)	48.1 (15.12)	0.015	40.4 (23.69)	49.8 (16.62)	0.066^b^
Sperm morphology (%)	65.1 (24.97)	79.2 (14.80)	0.008	63.0 (28.30)	77.3 (10.86)	0.009^b^

^a^Standard deviation.

^b^Paired *t*-test.

**Table 5:** Erectile function between Tualang honey and Tribestan.

Variable	ANOVA				ANCOVA			
	Mean (SD^a^)		*F* stat^b^	*P* value	EMM^c^ (95% CI^d^)		*F* stat^b^	*P* value
	Tualang honey	Tribestan			Tualang honey	Tribestan		
IIEF-5 score	20.6 (2.81)	21.9 (2.96)	3.44	0.068	20.7 (19.94, 21.43)	21.6 (20.87, 22.42)	3.42	0.070

^a^Standard deviation.

^b^ *F* statistic.

^c^Estimated marginal mean.

^d^Confidence interval.

**Table 6:** Hormonal profile between Tualang honey and Tribestan.

Variable	ANOVA				ANCOVA			
	Mean (SD^a^)		*F* stat^b^	*P* value	EMM^c^ (95% CI^d^)		*F* stat^b^	*P* value
	Tualang honey	Tribestan			Tualang honey	Tribestan		
LH (IU/L)	4.4 (1.87)	4.8 (2.19)	0.73	0.397	4.4 (3.69, 5.07)	4.8 (4.09, 5.52)	0.72	0.400
Testosterone (nmol/L)	14.8 (5.64)	14.5 (5.74)	0.34	0.565	15.0 (13.60, 16.32)	14.3 (12.89, 15.69)	0.47	0.496
FSH (IU/L)	6.7 (3.48)	7.2 (4.19)	0.94	0.337	7.1 (6.48, 7.67)	6.8 (6.16, 7.39)	0.49	0.488

^a^Standard deviation.

^b^ *F* statistic.

^c^Estimated marginal mean.

^d^Confidence interval.

Normal value: LH, luteinizing hormone 1–12 IU/L; testosterone, testosterone hormone 6–30 nmol/L; FSH, follicular stimulating hormone 1–12 IU/L.

**Table 7:** Safety profiles.

Variable	Estimated marginal mean (95% CI^a^)				*F* stat^b^	*P* value
	Tualang honey		Tribestan			
	Preintervention	Postintervention	Preintervention	Postintervention		
Haematological profile						
Hb	153.6 (149.50, 157.61)	153.0 (145.62, 160.33)	150.3 (146.13, 154.49)	143.4 (135.84, 151.00)	2.34	0.131
TWC	7.2 (6.53, 7.77)	7.2 (658, 7.84)	7.4 (6.73, 8.01)	7.4 (6.77, 8.07)	0.00	0.967
Platelet	264.3 (247.93, 280.66)	264.8 (244.44, 285.09)	248.6 (231.69, 265.43)	254.7 (233.76, 275.67)	0.45	0.505
Urea	4.5 (4.21, 4.86)	4.7 (4.29, 5.05)	4.5 (4.15, 4.82)	4.8 (4.37, 5.15)	0.33	0.567
Renal profile						
Sodium	136.6 (131.24, 142.06)	139.7 (139.15, 140.26)	140.4 (134.83, 145.98)	140.2 (139.58, 140.73)	0.78	0.381
Potassium	4.3 (4.14, 4.47)	4.2 (4.02, 4.32)	4.3 (4.12, 4.45)	4.2 (4.08, 4.40)	0.43	0.513
Creatinine	89.0 (83.83, 94.11)	88.1 (83.67, 92.44)	84.7 (79.41, 90.01)	89.5 (85.01, 94.05)	3.54	0.065
Uric acid	0.4 (2.36, 2.42)	0.4 (0.36, 0.43)	0.4 (0.36, 0.42)	0.4 (0.38, 0.45)	2.29	0.135
Liver profile						
Albumin	46.9 (44.99, 48.89)	45.7 (44.92, 46.55)	46.4 (44.43, 48.45)	45.5 (44.7, 46.34)	0.04	0.844
Globulin	31.2 (29.59, 32.74)	31.3 (30.12, 32.41)	31.9 (30.28, 33.53)	31.1 (29.92, 32.73)	0.65	0.425
T. bilirubin	10.4 (8.67, 12.21)	10.9 (9.24, 12.64)	10.8 (8.92, 12.58)	10.7 (8.91, 12.41)	0.35	0.558
ALP	71.2 (64.27, 78.20)	69.0 (62.43, 75.57)	73.3 (66.10, 80.46)	70.0 (63.23, 76.77)	0.14	0.712
ALT	43.3 (34.08, 52.57)	40.6 (31.75, 49.42)	45.9 (36.38, 55.43)	46.7 (37.58, 55.80)	0.85	0.360
AST	30.6 (26.03, 35.27)	28.7 (24.09, 33.33)	33.4 (28.61, 38.14)	32.5 (27.71, 37.23)	0.17	0.682

^a^Confidence interval.

^b^F statistic.

Normal value: Hb, haemoglobin 130–180 g/L; TWC, total white cells 4.0–11.0 × 10^9^/L; platelet 150–450 × 10^9^/L; sodium 135–145 mmol/L; potassium 3.5–5.1 mmol/L; creatinine 50–116 umol/L; uric acid 0.18–0.47 mmol/L; albumin 35–50 g/L; globulin 20–39 g/L; T. bilirubin, total bilirubin <21 umol/L; ALP, alkaline phosphatase 30–150 U/L; ALT, alanine transaminase <51 U/L; AST, aspartate transferase <41 U/L.
